# The Smc5-Smc6 Complex Regulates Recombination at Centromeric Regions and Affects Kinetochore Protein Sumoylation during Normal Growth

**DOI:** 10.1371/journal.pone.0051540

**Published:** 2012-12-20

**Authors:** Vladimir Yong-Gonzales, Lisa E. Hang, Federica Castellucci, Dana Branzei, Xiaolan Zhao

**Affiliations:** 1 Molecular Biology Program, Memorial Sloan-Kettering Cancer Center, New York, New York, United States of America; 2 Programs in Biochemistry, Cell and Molecular Biology, Weill Cornell Graduate School of Medical Sciences, New York, New York, United States of America; 3 IFOM, FIRC Institute of Molecular Oncology, Milan, Italy; University of Minnesota, United States of America

## Abstract

The Smc5-Smc6 complex in *Saccharomyces cerevisiae* is both essential for growth and important for coping with genotoxic stress. While it facilitates damage tolerance throughout the genome under genotoxin treatment, its function during unperturbed growth is mainly documented for repetitive DNA sequence maintenance. Here we provide physical and genetic evidence showing that the Smc5–Smc6 complex regulates recombination at non-repetitive loci such as centromeres in the absence of DNA damaging agents. Mutating Smc6 results in the accumulation of recombination intermediates at centromeres and other unique sequences as assayed by 2D gel analysis. In addition, *smc6* mutant cells exhibit increased levels of Rad52 foci that co-localize with centromere markers. A *rad52* mutation that decreases centromeric, but not overall, levels of Rad52 foci in *smc6* mutants suppresses the nocodazole sensitivity of these cells, suggesting that the Smc6-mediated regulation of recombination at centromeric regions impacts centromere-related functions. In addition to influencing recombination, the SUMO ligase subunit of the Smc5–Smc6 complex promotes the sumoylation of two kinetochore proteins and affects mitotic spindles. These results suggest that the Smc5–Smc6 complex regulates both recombination and kinetochore sumoylation to facilitate chromosomal maintenance during growth.

## Introduction

The evolutionarily conserved Smc5 and Smc6 proteins are members of the Structural Maintenance of Chromosomes (SMC) family [1]–[3]. In budding yeast, they bind six other proteins, termed non-SMC elements (Nse1–6), to form the Smc5–Smc6 complex [4], [5]. This complex is required for both normal growth and resistance to DNA damaging agents [5]–[9]. Under DNA damaging conditions, it facilitates replication throughout the genome [10], [11]. One of its functions is to prevent the accumulation of recombination intermediates generated during perturbed replication, presumably through replication restart or ssDNA gap filling [12]–[16]. These recombination intermediates are detected as X-shaped DNA structures by 2-dimensional gel (2D gel) analysis, and can be toxic to the cell. Indeed, the removal of several recombination proteins, such as the strand exchange protein Rad51, improves the survival of mutants of the Smc5–Smc6 complex under genotoxic stress [14], [15]. It has been proposed that this complex can promote the resolution of recombination structures under these conditions [10], [12]–[17]. In addition, the Smc5–Smc6 complex also influences early steps of recombination, such as regulating the DNA association of the key recombination mediator protein Rad52 at stalled replication forks [18].

How the Smc5–Smc6 complex contributes to genome maintenance pathways during normal growth is less clear. In budding yeast, several studies have implicated this complex in the maintenance of repetitive DNA sequences, particularly the rDNA locus where it is enriched [5], [8], [16], [19]–[21]. In specific mutants of this complex, replication and segregation of rDNA or its neighboring DNA, but not other genomic loci, are particularly defective [8], [20]. Because these defects are largely unaffected by the removal of Rad52 [8], [20], the main function of the Smc5–Smc6 complex at this repetitive locus during growth appears largely independent of recombination, unlike the situation under replication stress. On the other hand, removal of Rad52, Rad51, and other recombination proteins can improve the growth of *smc5* and *smc6* mutants and even suppress the lethality of their null alleles [9], [14], [15]. These results suggest that the Smc5–Smc6 complex likely regulates recombination at other genomic loci during growth.

To test this idea, we examined whether the Smc5–Smc6 complex affects recombination at centromeric regions. Similar to rDNA, centromeres and the surrounding ∼25 kb regions on all chromosomes are enriched with this complex, as assayed by genome-wide ChIP analysis [19]. Unlike rDNA, the association of the Smc5–Smc6 complex with these centromeric regions requires Scc2–Scc4, similar to other regions on chromosomal arms [19]. Centromeric regions are critical for chromosomal stability and inheritance. They provide special chromatid structures, such as intra-chromatid cohesion, and allow the assembly of kinetochores, which are large protein complexes that connect to microtubules and enable chromosome segregation in mitosis [22], [23]. While a great deal is known about these aspects of centromeric regions, how recombination influences these loci has rarely been addressed. As DNA replication is often stalled at centromeric regions due to kinetochore blockage, proper regulation of recombination during these events could be important for the stability of these regions [24], [25]. Here, we examine how the Smc5–Smc6 complex influences these putative recombinational events at centromeres, using both 2D gel analysis to detect recombination intermediates, and live cell imaging to reveal the localization of recombination foci at centromeric regions. Results obtained using these two methods show that Smc6 is required to suppress recombination intermediates and modulate recombination events at centromeric regions. We also provide genetic evidence suggesting that these roles impact centromere related functions. In addition, our data show that a similar type of regulation may also occur at other unique, non-centromeric regions.

The stability of many genomic loci is influenced not only by DNA metabolism but also by the functions of associated protein factors. In this regard, centromeric sequences are uniquely complex due to the binding of more than 60 kinetochore proteins. The assembly and dynamics of kinetochore proteins are highly regulated by protein modifications, including sumoylation [26], [27]. Because the Nse2/Mms21 subunit (referred to as Mms21 hereafter) of the Smc5–Smc6 complex is a SUMO (small ubiquitin like modifier) ligase that promotes the addition of SUMO to substrates [5], [28], [29], we tested the potential role of this complex in the sumoylation of kinetochore proteins. We show that Mms21 regulates the sumoylation of specific kinetochore proteins and affects spindle function. These results thus suggest for the first time that the Smc5–Smc6 complex can combine recombinational repair with kinetochore protein regulation to promote chromosomal maintenance during growth.

## Results

### *smc6–56* mutant cells contain increased levels of recombination intermediates at centromeric and other non-repetitive sequences

**Figure 1:**
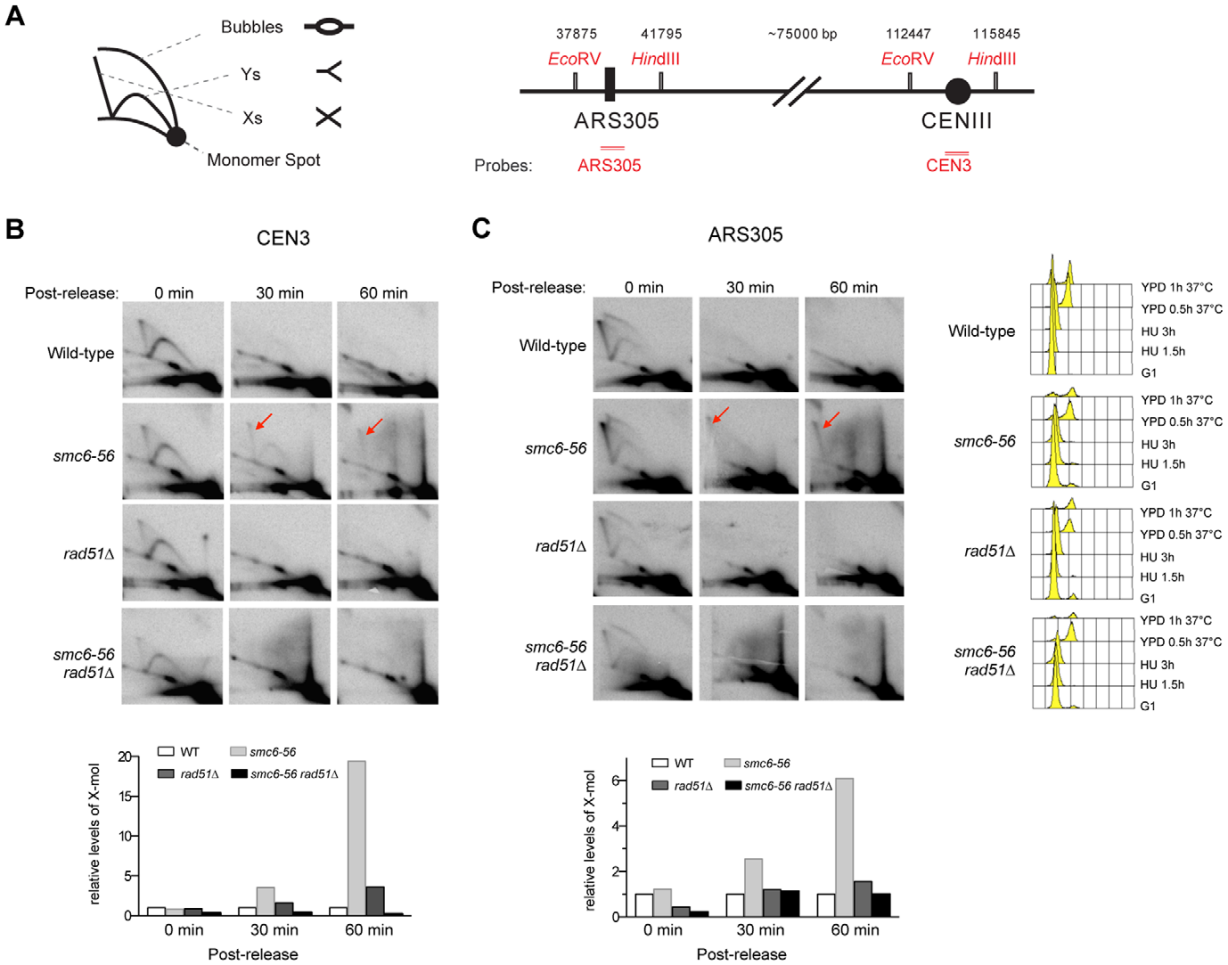
*smc6-56* cells accumulate recombination intermediates at centromeric and ARS305 sequences. (**A**) Schematics of 2D gel and genomic regions containing ARS305 and CEN3 sequences. The numbers above the genomic region are base pair coordinates from the left end of chromosome III. (**B–C**) Cells were arrested in G1 using alpha-factor, and synchronized in S phase using 0.2 M HU for 3 hours at 25°C. Cells were then washed and released into YPD medium at 37°C. Samples before and after release at indicated time points were examined by 2D gel analysis. Membranes were hybridized to a probe specific for the centromeric sequence on chromosome III (B) and another specific for ARS305 (C). FACS analysis before and after release is presented on the right panel in (C). Quantification of X-molecules (red arrows) is shown in the bottom panels. For both loci, the level of X-molecules increases in *smc6-56* cells compared with wild-type, and *rad51Δ* suppresses these increases.

To address the question of whether the Smc5–Smc6 complex affects the metabolism of recombination intermediates in the absence of DNA damaging agents, we used 2D gel electrophoresis to examine these structures in wild-type cells and cells harboring a chromosome-integrated, temperature sensitive, *smc6-56* allele [6]. We arrested both types of cells in G1 phase at permissive temperatures (of *smc6-56*) and further synchronized them in early S phase by HU (hydroxyurea) treatment, which inhibits dNTP production. Then the cells were released into normal media, allowing for replication at the non-permissive temperature of 37°C. Under this condition, wild-type and *smc6-56* cells resumed replication as determined by the appearance of 2N DNA peaks in the FACS profiles and the disappearance of replication intermediates on 2D gels (Figs. 1A, 1B, 1C and data not shown). Using a probe recognizing the centromeric sequence on chromosome III, we detected increased levels of X-shaped DNA molecules in *smc6-56* cells compared with wild-type cells at 30 and 60 minutes after release (Figs. 1A, 1B). These molecules were not observed in the absence of Rad51, confirming that they represent recombination intermediates likely formed between sister chromatids (Fig. 1B). Thus, *smc6-56* cells accumulate recombination intermediates at centromeric regions in the absence of exogenous genotoxins.

To examine if increased levels of recombination intermediates under these conditions are specific to centromeric regions, we examined ARS305, an early replication origin located about 75 kb distal to the centromere on chromosome III (Fig. 1A). Increased levels of recombination intermediates in this region were also detected in *smc6-56*, but not in *smc6-56 rad51Δ*, cells at 30 and 60 minutes after release into normal media (Fig. 1C). These results demonstrate that *smc6-56* cells contain increased levels of recombinational structures at both centromeric and other unique sequences during growth.

### Rad52 foci levels increase at centromeric and non-centromeric regions in *smc6-56* cells

**Figure 2:**
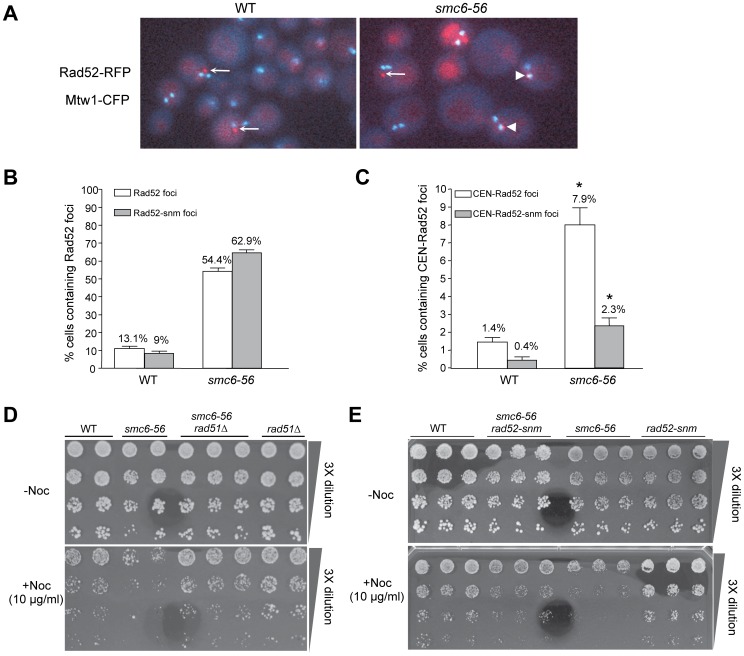
*rad52-snm* suppresses the increased repair foci at centromeric regions and nocodazole sensitivity of *smc6-56* cells. (**A–C**) *smc6-56* displays increased levels of Rad52 foci at centromeric and non-centromeric regions. (A) Representative overlay images of Rad52-RFP and Mtw1-CFP for wild-type (WT) and *smc6-56* cells. Arrowheads and arrows indicate the Rad52 foci that do and do not co-localize with Mtw1 foci, respectively. (B) Quantification of cells containing Rad52-RFP or Rad52-snm-RFP foci in wild-type and *smc6-56* cells. (C) Quantification of cells containing Rad52-RFP or Rad52-snm-RFP foci that co-localize with Mtw1-CFP foci. The difference between the two numbers labeled by the asterisks is statistically significant (p<0.01). (**D–E**) *rad51Δ* and *rad52-snm* rescue the nocodazole sensitivity of *smc6-56*. 2–3 different spores for each genotype were examined.

We next examined the levels of RFP-tagged Rad52 foci globally and at centromeric regions. This cytological approach assesses recombination events at a whole cell level [30] and complements the 2D gel analysis that assays specific sequences. Wild-type and *smc6-56* cells were shifted to 37°C for 4 hours before examination. Consistent with previous reports, about 13% (46/351) of wild-type cells contained Rad52 foci ([30]; Fig. 2A and white bar in Fig. 2B). By contrast, 54% (86/203) of *smc6-56* cells contained Rad52 foci, which is a 3.2-fold increase over wild-type levels (Fig. 2A and white bar in Fig. 2B).

We then examined how often Rad52 foci co-localized with centromere markers. Budding yeast centromeres and kinetochore proteins form one to two foci due to the clustering of centromeres [22]. Thus, live cell imaging of CFP-tagged kinetochore proteins, such as Mtw1, permits the assessment of colocalization of Rad52 foci with centromeric regions. We found that 1.4% (5/351) of wild-type cells contained Mtw1-colocalized Rad52 foci, which are referred to as CEN-Rad52 foci (Fig. 2A and white bars in Fig. 2C). About 8% (16/203) of *smc6-56* cells contained CEN-Rad52 foci, a 5.6-fold increase over wild-type levels (Fig. 2A and white bars in Fig. 2C). Taken together, the above cell biological results are consistent with 2D gel analysis (Fig. 1), and suggest that recombinational repair increases in frequency and/or takes longer at both centromeric and other chromosomal loci in *smc6-56* cells.

### *rad52-snm* suppresses nocodazole sensitivity and the increased level of recombination foci at centromeric regions in *smc6-56* cells

To assess whether the Smc6 regulation of recombination is biologically important at centromeric regions, we first examined whether alleviating the burden of recombination intermediates in *smc6-56* cells could suppress centromere-related defects. A surrogate readout of these defects is sensitivity to the microtubule and spindle destabilization drug nocodazole, which sensitizes mutants defective in centromere and kinetochore functions. Interestingly, we found that *smc6-56* mutants exhibited nocodazole sensitivity and that *rad51Δ*, which prevents the formation of recombination intermediates in *smc6-56* cells (Fig. 1B, 1C), suppressed this sensitivity (Fig. 2D).

As *rad51Δ* eliminates recombination throughout the genome, we next asked whether a recombination mutant that affects centromere recombination influences *smc6-56* nocodazole sensitivity. Various alleles of the key recombination protein Rad52 exhibit specific effects on different recombination processes [31]. One allele, *rad52-snm* (defective in Rad52 sumoylation, *K43R,K44R,K253R*) generally supports recombination functions in wild-type cells, but rescues the lethality of cells lacking the DNA helicases Sgs1 and Rrm3 [32], [33]. *sgs1Δ* cells accumulate recombination intermediates in the replication-blocking agent MMS (methyl methane sulfonate) similar to *smc6-56* [12], [34]. Though the mechanism of the *rad52-snm* suppression of *sgs1Δ* is not well understood, the similar defects of *sgs1Δ* and *smc6-56* suggest that *rad52-snm* may also influence recombination in the latter. Thus, we tested whether *rad52-snm* affects the levels of Rad52 foci in *smc6-56* cells grown at high temperatures.

As shown in Figure 2B, 9% (22/244) of *SMC6* cells contain Rad52-snm foci, compared with 62.9% (161/256) of *smc6-56* cells (grey bars in Fig. 2B). The increase in *rad52-snm* foci in *smc6-56* cells is similar to (though stronger than) that of Rad52 foci (white bars in Fig. 2B). Next, we determined the number of cells that exhibited colocalization between Mtw1 and *rad52-snm* foci. We found that 0.4% (1/244) *SMC6* and 2.3% (6/256) *smc6-56* cells contained these foci (grey bars in Fig. 2C). Chi-square tests show that the difference in the percentage of cells containing CEN-Rad52 foci vs. CEN-Rad52-snm foci is statistically significant for *smc6-56* cells (p<0.01). Taken together, these observations suggest that *rad52-snm* reduces the levels of Mtw1-colocalized, but not overall, Rad52 foci in *smc6-56* cells.

If the Smc6 regulation of recombination is biologically relevant at centromeres, *rad52-snm*, which specifically reduces the levels of recombination foci at centromeres in *smc6-56* cells, may influence the nocodazole sensitivity of these cells as seen for *rad51Δ*. Indeed, *rad52-snm* improved the growth of *smc6-56* cells on medium containing nocodazole (Fig. 2E). We interpret these results to mean that nocodazole sensitivity in *smc6-56* cells is partly due to impairment of recombination at centromeric regions, and that these recombination events are regulated by Rad52 sumoylation.

### *rad52-snm* does not affect recombination intermediate levels in *smc6-56* cells

**Figure 3:**
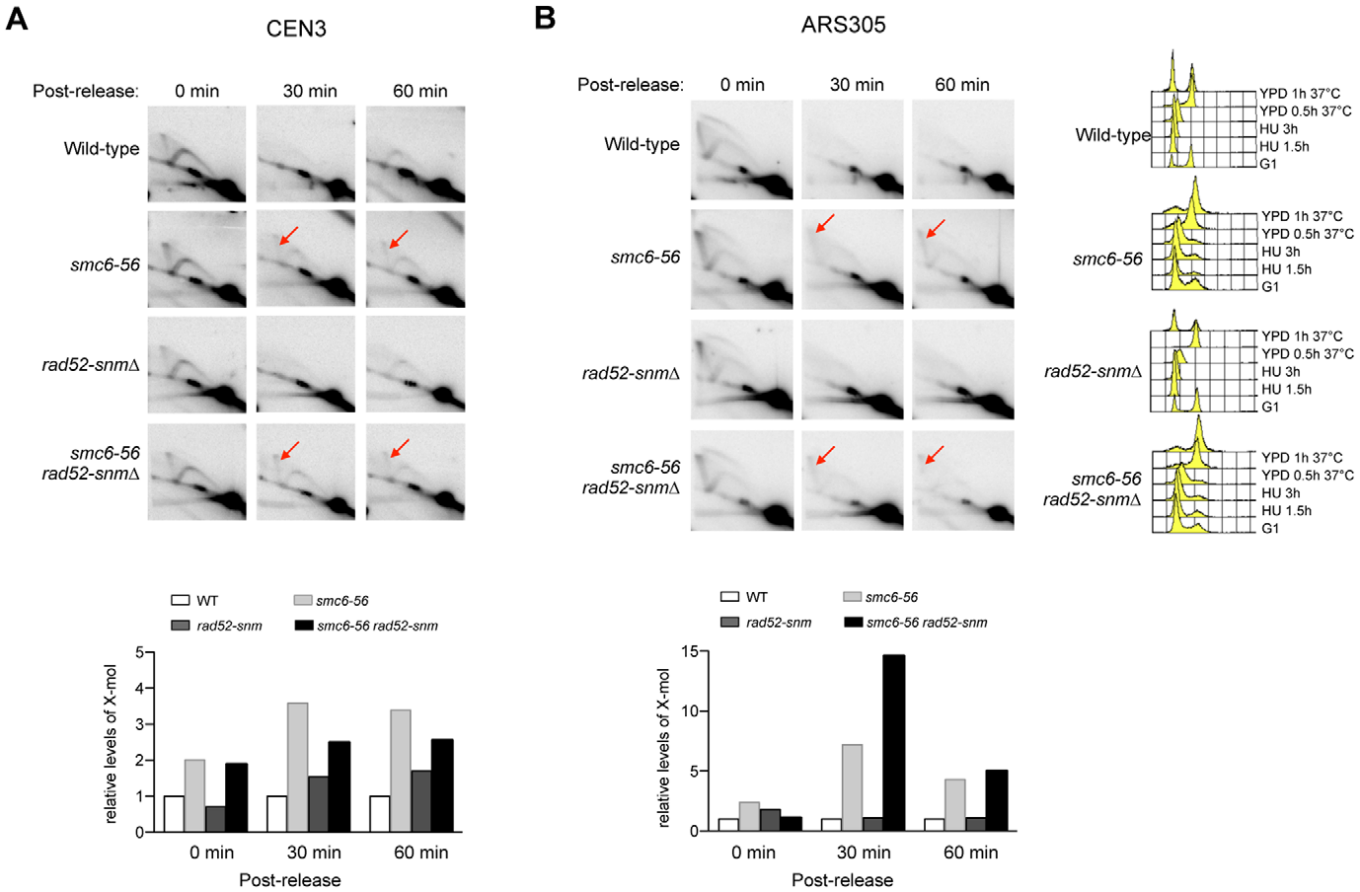
*rad52-snm* does not affect recombination intermediate levels. (**A–B**) Experiments were performed and analyzed as described in Figure 1. *rad52-snm* does not influence the levels of recombination intermediates (red arrows) in either wild-type or *smc6-56* cells at both CEN III (**A**) or ARS305 (**B**).

As mentioned above, how *rad52-snm* affects recombination is not fully understood. Its similarity with *rad51Δ* in suppressing the nocodazole sensitivity of *smc6-56* raised the possibility that *rad52-snm* may affect recombination intermediate levels. To test this idea, we performed 2D gel analysis using the same experimental scheme as described above (Fig. 1). *rad52-snm* cells behaved like wild-type, consistent with the notion that this mutation does not grossly affect recombination (Fig. 3). *rad52-snm* did not suppress the increased recombination intermediate levels in *smc6-56* cells at both CEN III and ARS305 loci (Fig. 3). These results suggest that *rad52-snm* affects recombination in a different manner than *rad51Δ*. As both suppressed the nocodazole sensitivity of *smc6-56*, *rad52-snm* likely alleviates a recombination defect distinct from recombination intermediate resolution. Considering the additional role of Smc5 and 6 in early recombination steps [18], it is possible that their regulation of both early and late steps of recombination contributes to nocodazole resistance.

### *mms21-11* and *smc6-56* decrease the sumoylation of two kinetochore proteins

**Figure 4:**
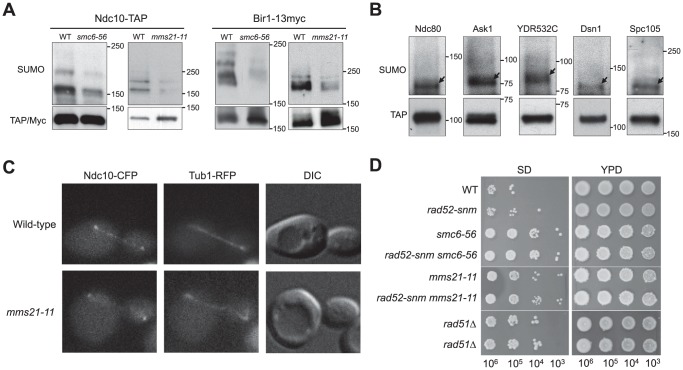
*mms21-11* and *smc6-56* affect sumoylation of specific kinetochore proteins, Ndc10 localization, and chromosome loss. (**A**) Sumoylation of Ndc10 and Bir1 is reduced in *mms21-11* and *smc6-56* cells. The indicated proteins are tagged at their own chromosomal loci. Sumoylation of these proteins was examined using an anti-SUMO antibody (top panel). The unmodified proteins were detected by anti-TAP or anti-Myc antibody (bottom panel). The slightly different appearance of the SUMO bands is due to different gel percentages. (**B**) Sumoylation of kinetochore proteins. Sumoylation of each indicated kinetochore protein was examined as in (A). SUMO forms of the proteins are indicated by arrows and migrate at positions approximately 20 kD above the unmodified proteins. (**C**) Ndc10 spindle localization is defective in *mms21-11* cells. Representative anaphase cells containing chromosomally tagged Ndc10-CFP and Tub1-RFP are shown. Note that Ndc10 is found in kinetochores and along spindles in wild-type cells. The spindle localization of Ndc10, but not the kinetochore localization, is defective in *mms21-11*. (**D**) *mms21-11* and *smc6-56* cells exhibit increased loss of chromosomes. Independent isolates of diploid strains were mated with haploid tester strains, and mating products from the indicated number of cells were selected on the SD (synthetic depleted) medium. YPD plates permit the growth of all cells regardless of mating status.

The results so far show that the Smc5–Smc6 complex regulates the levels of recombination intermediates and Rad52 foci at centromeres and other genomic loci, and that these effects likely pertain to centromere-related functions. As the Smc5–Smc6 complex contains a sumoylation enzyme subunit, Mms21, we tested whether this complex can also affect the protein components of the centromeres. Recent work has shown that sumoylation regulates kinetochore and spindle functions. Specifically, Ndc10 and Cep3, subunits of the Centromere Binding Factor 3 (CBF3), and Bir1 and Sli15, subunits of the chromosome passenger complex (CPC) were found to be sumoylated [26], [35]. We first examined whether their sumoylation levels were affected in *mms21-11*, a mutant lacking the SUMO ligase domain of Mms21 [5]. We found that Ndc10 and Bir1 sumoylation was diminished in *mms21-11* cells, while Cep3 and Sli15 sumoylation levels showed no changes (Fig. 4A and data not shown). The sumoylation of Ndc10 and Bir1 was similarly decreased in *smc6-56* cells (Fig. 4A). The co-depletion of sumoylation for these two proteins is consistent with a previous report showing that Bir1 sumoylation depends on Ndc10 sumoylation [26].

To test if additional kinetochore proteins are sumoylated in an Mms21-dependent manner, we examined sixty other kinetochore and spindle proteins tagged with the TAP module (Table 1; [36], [37]). We confirmed the modification of two linker kinetochore proteins (Ndc80 and Mcm21 [26]), and identified four new substrates: the outer kinetochore protein Ask1, and three linker kinetochore proteins, YDR532C, Dsn1 and Spc105 (Fig. 4B). As ∼17% (10/64) of tested kinetochore and spindle proteins are sumoylated, higher than the overall 8% of yeast proteins estimated to be sumoylated by proteomic studies [35], [38]–[42], there appears to be an enrichment of sumoylated substrates in these functional categories. Among all the sumoylated proteins identified here, *mms21-11* strongly affected the sumoylation of only Ndc10 and Bir1 (Fig. 4A and data not shown).

**Table 1:** Proteins examined for sumoylation.

Subcomplexes/functions	Sumoylated proteins	Non-sumoylated proteins
DAM1/DASH	Ask1	Dam1, Duo1, Dad1–4, Spc19, Spc34, Hsk3
CPC	Sli15, Bir1	Ipl1
CTF19/COMA	Mcm21	Ctf3, Ctf19, Okp1, Ame1, Mcm16, Mcm22, Mcm19, Chl4, Nkp1–2
SPC105	Spc105, YDR532C	
MTW1/MIND	Dsn1	Mtw1, Nnf1, Nsl1
NDC80	Ndc80	Spc24, Nuf2, Cnn1
CBF3	Ndc10, Cep3	Skp1, Ctf13
Motor proteins and Microtubules		Stu2, Kip3, Cin8, Bim1, Bik1, Kip1, Kar3, Tub1, Tub3, Tub4
Checkpoint		Mad1–3, Bub1–3, Dbf2, Mob1, Slk19, Sgo1
Others		Cbf1, Cse4, Scm3, Mif2, Rdh54

### *mms21-11* impairs the spindle localization of Ndc10

Ndc10 sumoylation has been shown to promote its localization to spindles without affecting its kinetochore localization [26]. Considering the effect of *mms21-11* on Ndc10 sumoylation, we tested whether this mutation affected Ndc10 spindle localization. While Ndc10 localized along the spindle in 95% (92/97) of wild-type anaphase cells, this localization was only observed in 69% (62/92, p<0.005) of *mms21-11* anaphase cells (e.g. Fig. 4C). *mms21-11* did not affect the kinetochore localization of Ndc10 (e.g. Fig. 4C). These results are consistent with the reported defect of a non-sumoylatable *ndc10* mutant [26], and provide evidence linking the sumoylation function of Mms21 to Ndc10 spindle localization.

### *mms21-11* and *smc6-56* lead to abnormal spindles and increased chromosome loss

**Figure 5:**
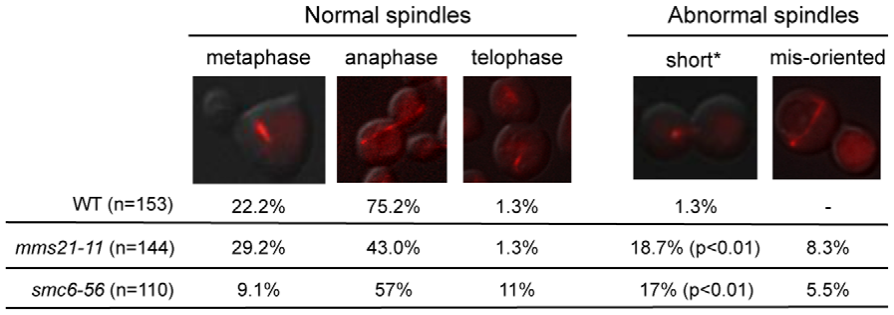
Spindle morphology in wild-type, *mms21-11*, and *smc6-56* cells. Spindle morphology was examined 75 minutes after cells were released from G1 arrest when the majority of cells were at anaphase. Only medium to large budded cells were counted. A representative picture is shown for each spindle category. Similar results were obtained for two strains of each genotype and the results of one pair are shown. Asterisk denotes large budded cells with short spindles. p value indicates that there is a statistically significant difference between wild-type and mutants.

The above results prompted us to examine whether *mms21-11* also affects mitotic spindle morphology. As shown in Figure 5, *mms21-11* cells contained spindles with abnormal morphologies, including mis-oriented (8.3%) and short spindles (18.7%), both of which were rarely seen in wild-type cells (Fig. 5). A similar defect was also observed in *smc6-56* cells. This result suggests that sumoylation by the Smc5-Smc6 complex influences spindle properties.

Next, we examined whether *mms21-11* elevates chromosome loss, which is often linked to spindle defects. Although increased loss of heterozygosity has been reported for *smc5* alleles [9], whether the sumoylation function of the Smc5–Smc6 complex affects chromosome loss has not been measured. We used a diploid bimating assay in which the loss of one copy of chromosome III (2N-1) allows cells to mate despite their diploid state [26], [43]. We spotted serial dilutions of mated cells to estimate the frequency of chromosome loss (see Methods). The chromosome loss frequency of wild-type diploids in this assay is similar to previous findings [26], [43]. *mms21-11* homozygous diploid strains exhibited about a 100-fold increase in this assay (Fig. 4D). A similar increase was seen for *smc6-56* cells at permissive temperature (Fig. 4D). We infer that sumoylation-dependent functions of the Smc5-Smc6 complex prevent chromosome loss. We observed that *rad52-snm* exhibited a normal level of chromosome loss and did not affect the levels in *smc6-56* or *mms21-11* cells (Fig. 4D). *rad51Δ* resulted in a similar level of chromosome loss as those of *mms21-11* and *smc6-56* (Fig. 4D), precluding the assessment of its possible suppression effect in this assay. While these results do not exclude the possibility that recombinational roles of the Smc5–Smc6 complex prevent chromosome loss, they raise the possibility that other functions of this complex, such as those involving kinetochore and spindle regulation, are important.

## Discussion

Here we examine how mutations of the Smc5–Smc6 complex affect recombination at centromeric sequences, kinetochore protein modification, spindle properties, and chromosome loss. 2D gel analysis provides physical evidence of increased levels of recombination intermediates at centromeric regions in *smc6-56* mutant cells (Fig. 1B). Consistent with this, live cell imaging shows that *smc6-56* cells contain increased levels of CEN-Rad52 foci (Fig. 2C). These two pieces of evidence suggest that Smc6 is required to regulate recombination at centromeric DNA and surrounding regions during growth. Since a sumoylation-defective *rad52* mutant that generates fewer centromeric foci can partially suppress the nocodazole sensitivity of *smc6-56* (Figs. 2C, 2E), recombinational roles of Smc6 at centromeric regions likely affect centromere-related functions. These data also suggest that recombinational repair at centromere regions involves a subpathway that entails both sumoylated Rad52 and the Smc5–Smc6 complex. A requirement for both has also been found in double strand break repair in rDNA, though *mms21-11* does not affect Rad52 sumoylation [32], [44]. These results provide the genetic bases for further examination of how the two collaborate in recombinational repair at both loci.

**Figure 6:**
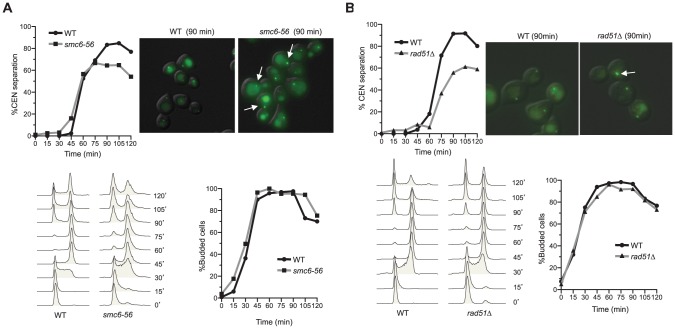
*smc6-56* and *rad51Δ* cells are defective in centromeric LacO array separation. (**A–B**) *smc6-56* and *rad51Δ* cells exhibit defects in centromere separation. Cells contain a LacO array integrated 12 kb distal to the centromere on chromosome IV. Cells were arrested in G1 at 23°C and then shifted to 37°C for 1 hour before release into the cell cycle at 37°C. Samples were taken every 15 minutes to examine cell cycle progression by FACS analysis (bottom left) and budding index (bottom right). These time points were also examined for centromere separation by microscopy (top left). The difference between the percentage of wild-type and *rad51Δ* or between that of wild-type and *smc6-56* cells containing separated GFP foci at 90, 105, and 120 min after release is statistically significant (p<0.01). Representative pictures at 90 minutes are shown (top right); arrows indicate cells with unseparated centromeres. Note that the background signals of LacI-GFP represent vacuolar staining.

The notion that recombinational roles of Smc6 are relevant to centromere function is also consistent with the previous observation that removal of the recombination protein Mph1 suppresses the centromeric separation defect of *smc6-56* [14]. This defect was initially thought to be unrelated to recombination, as *rad51Δ* did not suppress it [19]. However, we found that *rad51Δ* exhibited similar centromeric separation defects as *smc6-56* (Fig. 6A–6B). The reason for this defect is unclear, but may be due to the deleterious effect of eliminating multiple recombination sub-pathways. Since *rad51Δ* moderately suppresses the nocodazole sensitivity of *smc6-56* cells (Fig. 2D), the accumulation of recombination intermediates appears to be more deleterious than the lack of recombination.

Regulation of recombination by Smc6 is not restricted to centromeric regions. Increased levels of recombination intermediates and Rad52 foci were also detected in *smc6-56* cells at non-centromeric regions (Figs. 1C and 2B). Such a general role fits with the presence of the Smc5–Smc6 complex at many chromosomal arm regions [19]. Although probing the physiological importance of such a role at non-centromeric regions is not the focus here, we speculate that other replication blockage sites likely require proper regulation of recombination by this complex. The effects on recombination by Smc6 during growth described here are reminiscent of those under replication stress caused by exogenous DNA damage [11]–[16]. This suggests that preventing the accumulation of toxic recombination structures is a crucial function of the Smc5–Smc6 complex both during growth and under DNA damage conditions. We therefore propose that the Smc5–Smc6 complex responds in a similar manner to multiple situations that create additional burden on the replication machinery, whether drug-induced or intrinsic to the nature of the locus. Thus, its function is not restricted to exogenous causes of replicative stress, but is likely to be an important component of the replication program. In agreement with this notion, the Smc5–Smc6 complex prevents sequence loss at telomeres and break-induced replication during growth, both of which can result from a failure to proper regulate recombination during replication [21], [45]. As *rad51Δ* and *rad52-snm* reduced the nocodazole sensitivity of *smc6-56* cells, but differently affected recombination intermediate levels on 2D gel, it appears that the Smc5–Smc6 complex can affect more than one recombination steps as previously proposed.

This work also suggests that the Smc5–Smc6 complex affects kinetochore protein function. Our survey of 64 kinetochore and spindle proteins revealed ten SUMO substrates, four of which were not previously known (Table 1 and Figs. 4A–4B). Only Ndc10 and Bir1 sumoylation was decreased by *mms21-11*, an allele lacking the SUMO E3 ligase domain of Mms21 (Fig. 4A and data not shown). In addition, *mms21-11* reduced the spindle localization of Ndc10 (Fig. 4C), consistent with the finding that non-sumoylatable *ndc10* eliminates this localization [26]. The less penetrating defect of *mms21-11* is likely due to its partial effect on Ndc10 sumoylation. As the Siz SUMO ligases also contribute to Ndc10 and Bir1 sumoylation, Mms21 may collaborate with them, though an indirect effect can not be excluded [26]. We do not expect that *mms21-11* and non-sumoylatable *ndc10* exhibit the same set of defects, because *mms21-11* affects sumoylation of other proteins [5], [46]. Short and mis-oriented spindles seen in *mms21-11* anaphase cells (Fig. 5) were not observed in non-sumoylatable *ndc10* cells [26], and are likely due to a combined defect in the sumoylation of multiple Mms21 substrates. As *smc6-56* exhibited similar defects as *mms21-11* in spindle morphology and Ndc10 and Bir1 sumoylation (Figs. 4A and 5), the *smc6-56* allele likely impairs the sumoylation function of the Smc5–Smc6 complex.

In summary, our results suggest that the Smc5–Smc6 complex affects both recombination and kinetochore protein function during growth. While this work does not delineate the molecular connections between these two effects, results here provide bases for further study of their interplay. As mutants of the Smc5–Smc6 complex in fission yeast also exhibit chromosomal loss and an increased sensitivity to a microtubule destabilization drug, the roles of this complex at centromeric regions may be conserved [47], [48]. It is highly plausible that the integration of recombinational regulation and sumoylation via the Smc5–Smc6 complex contributes to the maintenance of other genomic regions during normal growth and under genotoxic stress. Future work will be needed to elucidate the interplay as well as the biological influence of these dual functions in genome maintenance.

## Materials and Methods

### Yeast strains, plasmids, primers and genetic manipulations

Strains containing chromosomal TAP-tagged (Table 1) kinetochore proteins were obtained from Open Biosystems [37]. These tagged strains were crossed to *smc6-56* or *mms21-11* strains to generate the corresponding mutant derivatives. Other yeast strains are listed in Table 2; where applicable, a single representative of each genotype is listed. The LacO-array assay strain, *rad52-snm* strain, and the *RFP-Tub1* strain were kind gifts from Andrew Murray, Michael Lisby, and Kerry Bloom, respectively. To construct Mtw1-CFP, the following primers were used to amplify the CFP region on the pFA6a-CFP plasmid: tag-mtw1-cfp-F2 (ATTGAAGAGCCTCAATTGGATTTACTTGATGATGTGTTACGGATCCCCGGGTTAATTAA) and tag-mtw1-cfp-R1 (AAGGTTGGCTGGCTACAGGATTCGAATTTTACGAAGTACTGAATTCGAGCTCGTTTAAAC). The PCR products were then used to tag Mtw1 at its own chromosomal locus using standard yeast protocols.

**Table 2:** Yeast strains used in this study.

Name	Relevant Genotype	Source
X2123-2A	*smc6-56-13myc::HIS3* W303	[14]
X2761-3C	*rad51Δ::LEU2* W303	This work
X2761-2B	*rad51Δ::LEU2 smc6-56-13myc::HIS3* W303	This work
X1432-5a	*RAD52-YFP MTW1-CFP* W303	This work
X1432-5b	*RAD52-YFP MTW1-CFP smc6-56-13myc::HIS3* W303	This work
X1429-7d	*rad52-K43R,K44R,K253R-YFP MTW1-CFP smc6-56-13myc::HIS3* W303	This work
X1429-13d	*rad52-K43R,K44R,K253R-YFP MTW1-CFP* W303	This work
X2050-13B	*rad52-K43R,K44R,K253R smc6-56-13myc::HIS3* W303	This work
X1465-5D	*NDC10-TAP::HIS3 mms21-11::LEU2* S288C	This work
X1711-8C	*NDC10-TAP::HIS3 smc65-56* S288C	
T658-3	*NDC10-CFP::HPH ura3-1::mCherry-TUB1::URA3* W303	This work
T657-1	*NDC10-CFP::HPH ura3-1::mCherry-TUB1::URA3 mms21-11::LEU2* W303	This work
PWY93-3B	*BIR1-13Myc::KAN* W303	[52]
X1508-2A	*BIR1-13Myc::KAN mms21-11::LEU2* W303	This work
X1740-6D	*BIR1-13Myc::KAN smc6-56* W303	This work
X3049-1	*mms21-11::KAN/mms21-11::URA* W303	This work
X3042-2	*smc6-56-13myc::HIS/smc6-56-13myc::KAN* W303	This work
X4573-6	*smc6-56-13myc::HIS/smc6-56-13myc::KAN rad52-K43R,K44R,K253R*/*rad52-K43R,K44R,K253R* W303	This work
X4204-1	*rad52-K43R,K44R,K253R*/*rad52-K43R,K44R,K253R* W303	This work
X4571-7	*mms21-11::KAN/mms21-11::HIS rad52-K43R,K44R,K253R*/*rad52-K43R,K44R,K253R* W303	This work
X4209-6	*rad51Δ::LEU2/rad51Δ::LEU2* W303	This work
X2133-18c	*his3-11::pCUP1-GFP12-lacI12::HIS3 trp1-1::LacO::TRP1* W303	This work
X2133-14b	*his3-11::pCUP1-GFP12-lacI12::HIS3 trp1-1::LacO::TRP1 smc6-56-13myc::HIS3* W303	This work
X1991-1A	*his3-11::pCUP1-GFP12-lacI12::HIS trp1-1::LacO::TRP1* W303	This work
X2133-15D	*his3-11::pCUP1-GFP12-lacI12::HIS3 trp1-1::LacO::TRP1 rad51Δ::LEU2* W303	This work
X2065-28B	*rad51Δ::LEU2 smc6-56-13myc::HIS3* W303	This work

Strains in this study are either derivatives of W1588-4C, a *RAD5* derivative of W303 (*MAT* **a** *ade2-1 can1-100 ura3-1 his3-11,15 leu2-3,112 trp1-1 rad5-535* [53]), or in S288C background. Strain backgrounds are indicated.

### Cell imaging

All imaging was performed on an Axioimager microscope with a 100× objective lens (NA = 1.4). Cells were processed for microscopy as described previously [30], except that the exposure times used for each fusion protein are as follows: Rad52-RFP, 1s; Tub1-RFP, 1s; Mtw1-CFP, 1s; and LacO assay, 1s. In all cases, 14–18 Z-sections with a 0.5 µm step size were taken to cover the whole yeast cell and maximal projections are shown for all figures. For experiments examining asynchronous *smc6-56* cells, both mutant and wild-type cells were incubated at 37°C for 4 hours before imaging. For each measurement, at least two strains per genotype were tested. Statistical analysis of focus frequency was carried out using a Chi-Square test, and the p-values for each relevant comparison are indicated in the figure legends and tables.

### 2D gel analysis to examine recombination intermediates

Purification of DNA intermediates, 2D gel analysis, and quantification of replication intermediates were carried out as previously described [49]. The DNA samples were digested with *Eco*RV and *Hind*III and analyzed with probes recognizing CEN3 and *ARS305*. The following oligos were used to amplify ARS305 and CEN3 probes: ARS305-FW: GTTCCGAAACAGGACACTTAGC, ARS305-RV: ATCCAGGAGGGACTCAATGTAG, CEN3-FW: CCGAGAGAGCTGCAAAATTAG, CEN3-RV: GATTCTCACCGCATGACAAGTG. The PCR-generated CEN3 probe is ∼1 kb that contains the 118 bp centromeric sequence and ∼900 bp of surrounding sequence.

### Detection of sumoylated proteins

Strains containing TAP-tagged proteins involved in kinetochore and spindle functions (Table 1) were obtained from the collection of Open Biosystems [37]. Each strain was inoculated in YPD to mid-log phase and cell lysates were made under denaturing conditions to preserve sumoylated proteins as described [50]. Immunoprecipitation of the TAP tagged protein and detection of the sumoylated forms have been described previously [50]. Western blotting was performed according to standard procedures using the following primary antibodies: anti-Myc (9E10; Sigma), anti-TAP (P1291, Sigma) and anti-SUMO [5]. Detection was performed using Enhanced Chemiluminescence (GE Healthcare). Note that sumoylated forms normally comprise a small percentage of the proteins and are not detected by the anti-TAP antibody on the exposures shown.

### Centromere separation assays

This assay was performed as described in [51]. In brief, wild-type, *smc6-56* and *rad51Δ* cells were grown at 23°C to early log phase. Cells were arrested at G1 phase by the addition of alpha-factor at 23°C. Cells were then shifted to 37°C for 1 hour to inactivate the Smc5–Smc6 complex in the presence of alpha-factor and then released into normal media at 37°C. Samples were taken every 15 minutes to examine cell cycle progression by FACS analysis and budding index, and to examine centromere separation by microscopy. Alpha-factor was added back to the culture 45 minutes after release to minimize the number of cells entering the next cell cycle. For each measurement, at least two strains of each genotype were analyzed and produced consistent results; thus, results from one trial are presented. Statistical analysis of focus frequency was carried out using Chi-square tests and the p-values for relevant comparisons are indicated in the figure legends.

### Chromosomal loss assays

This assay was performed as described previously, with minor modifications [43]. In brief, 1 OD of diploid cells were mated with the same number of *MAT* **a** tester cells for 24 hours, and serial dilutions of mated cells were spotted onto YPD and SD (synthetic deplete) plates and incubated at 30°C for 36 hours before being photographed. When diploid cells lose one of the two copies of chromosome III, they gain the ability to mate with Mat **a** cells, resulting in growth on SD plates. More than 3 independent diploid clones were tested for each genotype.
